# Review on Multiple Facets of Drug Resistance: A Rising Challenge in the 21st Century

**DOI:** 10.3390/jox11040013

**Published:** 2021-12-13

**Authors:** Mousumi Saha, Agniswar Sarkar

**Affiliations:** 1 Department of Microbiology, Ballygunge Science College, University of Calcutta, 35, Ballygunge Circular Road, Kolkata 700019, India; 2 Virus Unit [NICED-ICMR], GB4-1st Floor, ID and BG Hospital, 57, S. C. Banerjee Road, Beliaghata, Kolkata 700010, India; ognish@gmail.com

**Keywords:** multidrug resistance, infection control strategy, ESKAPE, COVID-19, molecular mechanism, in-silico analysis

## Abstract

With the advancements of science, antibiotics have emerged as an amazing gift to the human and animal healthcare sectors for the treatment of bacterial infections and other diseases. However, the evolution of new bacterial strains, along with excessive use and reckless consumption of antibiotics have led to the unfolding of antibiotic resistances to an excessive level. Multidrug resistance is a potential threat worldwide, and is escalating at an extremely high rate. Information related to drug resistance, and its regulation and control are still very little. To interpret the onset of antibiotic resistances, investigation on molecular analysis of resistance genes, their distribution and mechanisms are urgently required. Fine-tuned research and resistance profile regarding ESKAPE pathogen is also necessary along with other multidrug resistant bacteria. In the present scenario, the interaction of bacterial infections with SARS-CoV-2 is also crucial. Tracking and in-silico analysis of various resistance mechanisms or gene/s are crucial for overcoming the problem, and thus, the maintenance of relevant databases and wise use of antibiotics should be promoted. Creating awareness of this critical situation among individuals at every level is important to strengthen the fight against this fast-growing calamity. The review aimed to provide detailed information on antibiotic resistance, its regulatory molecular mechanisms responsible for the resistance, and other relevant information. In this article, we tried to focus on the correlation between antimicrobial resistance and the COVID-19 pandemic. This study will help in developing new interventions, potential approaches, and strategies to handle the complexity of antibiotic resistance and prevent the incidences of life-threatening infections.

## 1. Introduction

Recently, antibiotic resistance in various bacteria has emerged as a global threat to the treatment options for bacterial infections, and a subject of current research [1,2]. Antibiotic resistance is not only a major complication: antimicrobial resistance (AMR) is also one of the top ten global public health concerns flourishing all over the world. Antibiotics, antiviral, antifungal, and antiparasitics are commonly known as antimicrobials. The scenario regarding the adverse effects of resistance is much bigger than its imagination. Antiviral drug resistance is also increasing at an alarming rate, and has become a concern for immunocompromised patients. Antiretroviral drugs are one of the best examples of this problem. Drug resistance to malaria parasites and fungal infections are increasing and infuriating the treatment situation. Dated back in May 2015, the WHO has developed Global Action Plan on AMR with five objectives to fight steps against AMR [3,4]. On 11 March 2020, WHO [5] declared that the world was under the calamity of a Coronavirus disease (COVID-19) pandemic caused by novel Severe Acute Respiratory Syndrome Coronavirus (SARS-CoV-2) [6,7,8]. Interestingly, in November of the same year, World Antimicrobial Awareness Week was arranged, and a slogan of ‘Antimicrobials Handle with Care’ was highlighted to win the battle of antibiotic resistance [9], as, in the present situation, antibiotic misuse or excessive use can be related to COVID-19 treatment [10]. Few environmental bacteria are also believed to acquire antibiotic resistance determinants, which could be due to co-existence/evolution or horizontal gene transfer (HGT) [11,12]. Thus, all of these factors, along with the excessive use of antibiotics, are responsible for the emergence of resistance in isolates [13]. Threats caused by both gram-negative and gram-positive resistant bacterial strains have compelled scientists to explore new classes of antibiotics and novel approaches to tackle the problem of resistance [14,15].

AMR is not only hindering the health sector but is also an economic burden for developed, as well as for developing countries [16]. Several treatment procedures are at risk without antibiotics, such as organ transplantation, chemotherapy, and surgeries. However, not only antibiotics are necessary for treating infectious diseases and small injuries. However, without common antibiotics, more expensive medicine, prolonging the duration of treatment and longer hospital exposure, will enhance the cost of treatment with the requirement of intensive care, and finally lead to life-threatening damage.

In this review, we attempted to embellish the key features of antibiotic resistance (AMR), its consequences, mechanisms, regulation, association, and control strategies. Raising awareness and educating people are the most important steps for controlling antibiotic resistance. Irradiation of AMR is a goal of sustainable development, and together we must achieve this. The present review focuses on the surveillance of the origin of antibiotic resistance, genetic and functional characterization of resistance factors, and therapeutic options. Through this review, we also attempted to draw attention to the irrational use of antibiotics and their negative consequences. The review will help researchers/scientists in creating preventive tools and diagnostic pathways. Here, we tried to explore the multiple interaction impact of AMR with COVID-19 by understanding the usage of antimicrobial agents, the role of the healthcare system, and preventive measures.

## 2. Brief History and Background

The discovery of penicillin in 1928 by Alexander Fleming was a pillar for the foundation of the modern era of treatment for bacterial infections [17]. The origin of antibiotics dates back a few decades, and since then they have been playing an important role in impeding the growth of pathogenic microbes. Antibiotic resistance emerged in the 1960s, when various enteric and other gram-negative bacteria developed resistance toward highly used drugs. Overall, three major elements of AMR development are emergence, transmission, and level of infection. For example, *Neisseria gonorrhoeae (N.* *gonorrhoeae*) developed resistance to ampicillin, and *Haemophilus* developed resistance to ampicillin, tetracycline, and chloramphenicol [18,19]. Similarly, sulfonamide resistance originated in the 1940s, and soon after the origin of sulfonamide resistance, pharmaceutical companies and researchers had to face another hurdle when aminoglycoside-resistant *S. aureus* came into existence. Afterwards, with the development of new antibiotics, such as methicillin, vancomycin, and fluoroquinolones, the emergence of resistance to these antibiotics also developed gradually; methicillin-resistant *S. aureus* (MRSA) strains are among the rapidly propagating bacterial strains [20].

With the advancement of genome biology, scientists are becoming aware of the genome sequence of various bacteria, and approximately 20,000 antibiotic resistance genes have been identified to date. However, the lack of data regarding molecular, genetic, and biochemical mechanisms is the major hindrance in resolving this vital issue [21]. Shortage of funds, as well as development of new drugs, are also the key concerns. The incorporation of bioinformatics tools and techniques can help to recover this hurdle with the available information of genome sequence [22]. Disposal of antibiotics or related items without adhering to rules and regulations, hospital-acquired antibiotic resistances, improper hygiene and sanitation systems, excessive utilization of antibiotics in livestock management (poultry, aquatic, etc.), and international travel are a few factors for the outbreak of antibiotic resistance in the human and animal community [1]. All of these factors contribute to the escalation of resistance in the environmental niche, which is also a serious global concern [11]. Examples of some infectious diseases are pneumonia, tuberculosis, gonorrhea, salmonellosis, foodborne disease, blood poisoning, etc., which are treated with different types of antibiotics [1,23]. However, with increased resistance, common antibiotics are becoming less functional for the treatment of various diseases. These problems are associated with prolonged treatment, enhanced medical cost, and, in some cases, finally result in mortality. Therefore, new drugs and different treatment options are required [9,24].

## 3. Features of Different Resistant Bacteria

The World Health Organization (WHO) and Center for Disease Control and Prevention (CDC) declared the rapidly growing antibiotic resistance as one of the major global concerns [25]. As per the CDC report (13 November 2019; www.cdc.gov/drugresistance/pdf/threatsreport/2019), antibiotic resistance threats were categorized under four sections, with the name of the strains as described here: (a) Urgent Threats (Carbapenem-resistant *Acinetobacter*; *Candida auris*; *Clostridioides difficile*; Carbapenem-resistant Enterobacterales; Drug-resistant *Neisseria gonorrhoeae),* (b) Serious Threats (Drug-resistant *Campylobacter;* Drug-resistant *Candida*; ESBL-producing Enterobacterales; Vancomycin-resistant *Enterococci* (VRE); Multidrug-resistant *Pseudomonas aeruginosa*; Drug-resistant nontyphoidal *Salmonella*; Drug-resistant *Salmonella* serotype Typhi; Drug-resistant *Shigella*; Methicillin-resistant *Staphylococcus aureus* (MRSA); Drug-resistant *Streptococcus pneumoniae*; Drug-resistant Tuberculosis), Concerning Threats (Erythromycin-Resistant Group A *Streptococcus*; Clindamycin-resistant Group B *Streptococcus*), and Watch List (Azole-resistant *Aspergillus fumigatus*; Drug-resistant *Mycoplasma genitalium*; Drug-resistant *Bordetella pertussis*).

Based on the extent of resistance towards different drugs, AMR has been further classified into multidrug-resistant (MDR), extensively drug-resistant (XDR), and pan drug-resistant (PDR) [26,27]. Recently, difficult-to-treat resistance (DTR) and modified DTR have also been categorized [25,28]. “MDR was defined as acquired non-susceptibility to at least one agent in three or more antimicrobial categories as per guidelines. XDR was defined as non-susceptibility to at least one agent in all but two or fewer antimicrobial categories listed and PDR was defined as non-susceptibility to all agents in all antimicrobial categories” according to the European Centre for Disease Control (ECDC) and the CDC [28]. *Staphylococcus aureus (S. aureus*), *Enterococcus* spp., *Enterobacteriaceae*, *Pseudomonas aeruginosa (P. aeruginosa*), and *Acinetobacter* spp. are just a few examples of multiple drug-resistant bacteria [27]. MDR is an obstacle for developing countries due to inadequate health systems, and limitations of funds and resources. MDR gram-negative bacteria such as extended-spectrum β-lactamase (ESBL)-producing *Enterobacteriaceae* and carbapenem-resistant *Enterobacteriaceae* (CRE) have become a health issue for neonates. Few specific treatment options and monitoring initiatives have been taken into account by WHO under NeoAMR Project [29]. Bacteria possess unique self-defence mechanisms against their antibiotics [30]. 

To understand the conception and consequences of MDR, the origin, source, mobilization, and molecular factors of these determinants should be thoroughly studied. MDR is becoming increasingly common among various pathogenic and non-pathogenic strains, and is mostly associated with the acquisition of genes and/or modification in the antibiotic target genes [31]. The regulators of MDR in bacteria are considered cardinal to the survival and adaptability of the bacterial community to unfavorable and extreme conditions, including the presence of antibiotics or other drugs. Antibiotic resistance or MDR is associated with the acquisition of genes and/or modification in the antibiotic target genes [32]. The accessibility of a large amount of sequencing information for bacterial genomes along with the functional data of various mobile genetic elements that have amassed over for nearly four decades has permitted the research community to broadly review the epidemiological aspects of MDR hereditary determinants of pathogenic and natural microbes [33].

## 4. Origin and Development of Resistance

Essentially, antibiotic resistance is developed by bacteria via their natural selection process or through their survival strategy in harsh environmental conditions, but irrational use has accelerated the resistance. Resistant bacteria then invade the human or animal and develop various infectious diseases [34]. Molecular studies on determining the origin strongly suggest that microorganisms are proficient in genetic recombination and may exchange antibiotic resistance-related gene cassettes. Comparative genomic and transcriptomic studies of the strains have helped researchers to assess their phylogenetic orientation and gene expression. However, the functional diversity and similarity between resistant strains should be explored to improve the modern healthcare system and reduce the incidence of community-acquired infections [21,35].

Several gram-negative bacteria are responsible for causing community-acquired infections and are thus considered hazardous, posing a risk to the modern healthcare system [1]. Integration of antibiotic resistance genes or MGEs is responsible for antibiotic resistance into bacterial chromosomes [36]. However, information regarding the mechanism and expression of the regulators of antibiotic resistance is scant. The consumption of antibiotics by animals in their food leads to some degree of antibiotic resistance even in human beings [1]. The utilization of animal models can help in assessing the effects of antimicrobial resistance and pathological mechanisms. With the advancement of science, various semi-synthetic, synthetic, and broad-spectrum antibiotics have been developed with specificity toward the causative agent and not the host. However, the outrageous use of antibiotics in the human, as well as animal healthcare systems, is one of the major factors for the emergence of MDR [26]. Lack of treatment guidelines, and over-prescription of antibiotics by health workers and veterinarians also contribute to in rising in antibiotic resistance [1].

**Figure 1:**
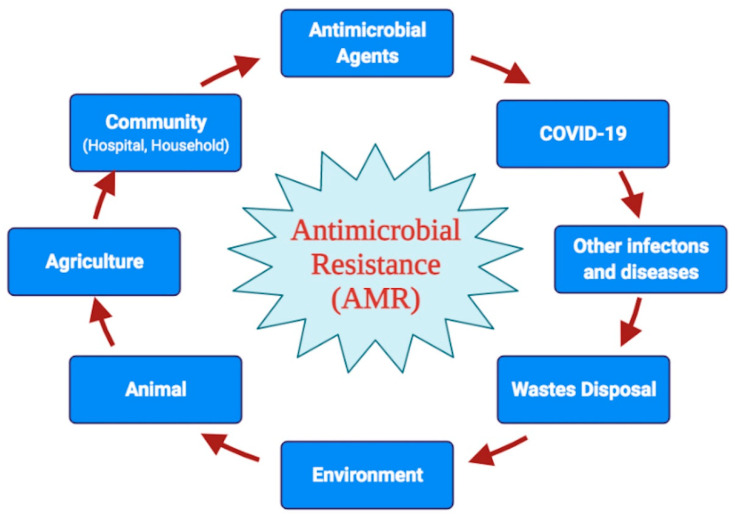
Schematic representation of various factors, causes and consequences contributing towards the emergence and spread of antimicrobial resistance.

Resistance toward a specific antibiotic drug may originate at any time point, and the reasons could be natural selection pressure or non-judicial use of antibiotics without adequate knowledge. The food chain may be another reason for the origin of drug resistance [37]. Typically, similar classes or types of antibiotics are used in agriculture, human treatment, as well as in cattle or dairy farms, that lead to the spread of antibiotic resistance followed by the transmission of resistance traits. Excessive use of antimicrobial agents in human, animal, and agricultural production leads to environmental contamination, especially water bodies, which need further consideration [1]. Colistin addition to animal feed needs to be restricted or stop to avoid antibiotic resistance and contamination [38]. The use of huge biocide-based items and their disposal into soil or water bodies may have some negative impact on the environment, and may elevate the rate of resistance traits among micro-organisms [39]. All of these reasons contribute to the origin, emergence, and spread of resistance from the environment, waste disposal and community (Figure 1).

## 5. Characterization of Antibiotic Resistance Factors

Antibiotic resistance is vital for the survival of bacteria, and they can acquire self-defence against antibiotics. Thus, antibiotic resistances are carried via natural selection as well as directly by the transfer of genes or genetic determinants. Excessive use of antibiotics or an incomplete course of antibiotics leads to increased antibiotic resistance (which can be strongly correlated with its consumption). Infectious or pathogenic bacteria remain unaffected or partially affected, and this may increase the chances of relapse of the infection or the disease. In these cases, similar or sometimes different broad-spectrum antibiotics are used, and thus bacteria may acquire MDR through different mechanisms. However, all these factors lead to the origin and occurrence of antibiotic resistance among clinical as well as environmental strains [8,24].

Strains of environmental origin may carry various mobile genetic elements (MGE), including antibiotic resistance genes [12,13]. The genetic exchange occurs extensively in the environment, as well as under in vivo conditions, and other opportunistic pathogens may inherit or uptake resistant genes. MGE in gram-negative and gram-positive bacteria are assumed to play a vital role in conferring adaptation in the bacterial community, permitting them to rapidly adapt to unfavorable conditions, including the presence of antibiotics [12,13]. Integrative and conjugative plasmids are effective means of scattering the MDR traits in a broad range of pathogenic *Enterobacteriaceae* strains. Studies have reported a vital role of versatile hereditary components, such as conjugative plasmids and integrative conjugative elements (ICEs) in the spread of MDR [12,40,41]. Therefore, extensive molecular characterization of these genes must be developed for novel therapeutic strategies.

Broadly, antibiotic resistance can be classified into four types: (1) natural/intrinsic resistance, and (2) acquired resistance. Table 1 summarizes a few mechanisms of resistance to different target drugs having different modes of action.

### 5.1. Natural/Intrinsic Resistance

Intrinsic resistance can be defined as the ability of a bacterium to resist the effect of an antibiotic due to its inherent properties. An excellent example of an intrinsic resistance mechanism is the absence of a susceptible target site for a particular antibiotic in *Pseudomonas*. Triclosan, a broad-spectrum biocide especially against gram-positive bacteria and several gram-negative bacteria, is incapable of preventing the growth of *Pseudomonas*. Initially, the resistance was supposed to be due to active efflux, but later on, the presence of *fabI* (insensitive allele) which encodes an extra enoyl-acyl carrier protein reductase enzyme, a target site for triclosan, was reported [46]. Similarly, lipopeptide daptomycin, approved in 2003 for clinical practice, is an active drug against gram-positive bacteria (36). However, it is ineffective against gram-negative bacteria. The cytoplasmic membrane of gram-negative bacteria contains the lesser proportion of anionic phospholipids than gram-positive bacteria. Thus, the efficacy of Ca^2+^ mediated daptomycin insertion into the cytoplasmic membrane is decreased [47]. Additionally, some antibacterial compounds that are unable to cross the outer membrane are considered as a means of intrinsic resistance. In gram-positive bacteria, vancomycin inhibits peptidoglycan crosslinking by targeting D-Ala-D-Ala peptides, whereas, in the case of gram-negative bacteria, vancomycin cannot pass through the outer membrane. The synthesis of β-lactamases (e.g., extended-spectrum β-lactamases [ESBLs] enzymes, carbapenemases, and metallo-β-lactamases) has reportedly increased the rate of acquired MDR infections, and it has particularly led to third-generation cephalosporin and carbapenem resistance. Notably, various genes associated with intrinsic resistance to different classes of antibiotics such as lactams, aminoglycosides, and fluoroquinolones in *P. aeruginosa*, *S. aureus*, and *Escherichia coli* (*E. coli*) have been identified through high-throughput tools of high-density genome mutant libraries [48]. Genes responsible for drug-resistant *Salmonella* and *E. coli* include *AmpC, bla-TEM-1, bla-CTX- M-15, VIM-1, NDM-1, floR, tetG*, and the *mcr-1* gene that encodes colistin drug resistance [49]. As an example, the inactivation of redundant *E. coli* genes enabled the identification of putative targets, such as FabI, SapC, RecQ, TrxA (thioredoxin), TrxB (thioredoxin reductase), DacA, and D-Ala-D-Ala carboxypeptidase, for susceptibility phenotyping. Inhibition of these genes may significantly stimulate the activity of drugs such as triclosan, rifampicin, aminoglycosides, nitrofurantoin, and some β-lactams. Bacteria may acquire antibiotic resistance through various other mechanisms, such as antibiotic efflux or poor drug penetration resulting in the reduction of the intracellular concentration of antibiotic, modification of the antibiotic target site due to posttranslational target modification or genetic mutation of the target, and inactivation of antibiotics through modification or hydrolysis [32,50]. Plasmid coding colistin-resistant (mcr-1 dependent) *E. coli* was first isolated from raw meat, animals, and humans in China [51]; Mcr-1 harboring *E. coli* was also reported in migratory birds and human clinical isolates in Pakistan and MDR (ESBLs, quinolones). *E. coli* was reported from poultry farms, wild birds, pigeons, dogs, and humans [52], as a few examples. *N. gonorrhoeae* has developed resistance to sulphonamides, penicillins, tetracyclines, macrolides, fluoroquinolones, and early generation cephalosporins [53]. Apart from rifampicin-resistant *Mycobacterium tuberculosis* (*M. tuberculosis*) strains also show multi-drug resistance and pose a major risk factor for the treatment of tuberculosis [54].

### 5.2. Acquired Resistance

Acquired resistance is the most important trait, and its mechanism can be further subdivided into the following types: (1) decrease uptake of the drug due to reduction in the internal or external membrane permeability or changes in the cytoplasmic membrane composition; (2) enzymatic inactivation or degradation of antibiotics; (3) active efflux pump system; (4) resistance associated with alteration of the target or change in the structure of the target, and; (5) in some cases, the use of alternative metabolic pathways serves as the resistance mechanism [22]. The gene or the genetic elements responsible for acquired resistance may be chromosomal or extrachromosomal. Antibiotic resistance can occur via various mechanisms linked with virulence factors, outer membrane proteins cell envelope factors specific enzymes, quorum sensing, and biofilm formation and protein secretory systems [22]. Studies have indicated that bacteria are prone to undergo genetic recombination, and thus exchange various virulent and antibiotic resistance-related gene cassettes. Comparative genetic and transcriptomic studies of resistant strains will reveal their phylogenetic orientation and gene expression that can help scientists to discover the resistance determinants in different enteric pathogens and environmental reservoirs of genetic elements. HGT facilitates the transmission of both resistance and virulence factors between bacterial genera or species [12]. Features such as MGEs and biofilms help bacteria survive and overcome the antibacterial activity of therapeutics [12].

**Figure 2:**
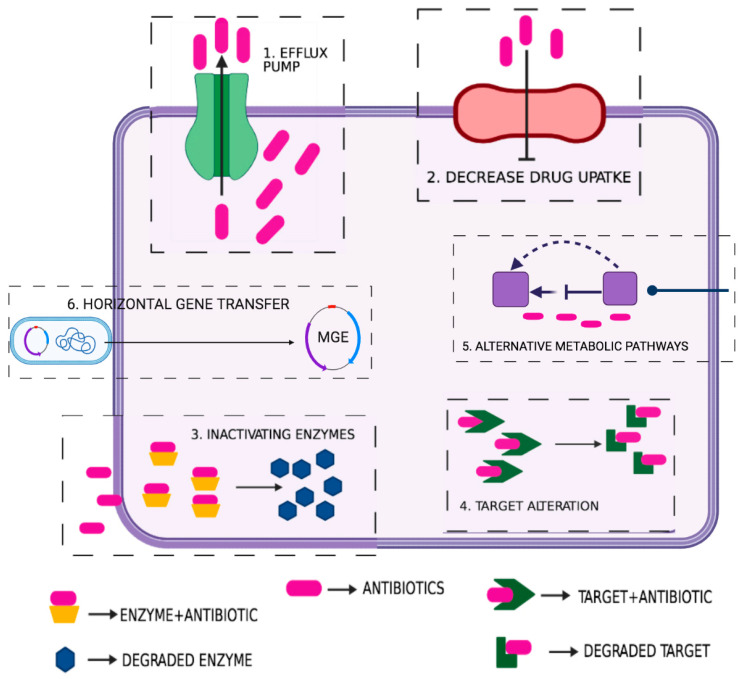
Diagrammatic illustration of few possible mechanisms in bacteria contributing towards various antibiotic resistance such as: 1. Active efflux of various antibiotic via pumps; 2. Prevention or decrease drug uptake by the cell; 3. Antibiotic inactivating enzymes; 4. Modification or alteration of targets; 5. Use of alternative or bypass metabolic pathways; 6. Acquired antibiotic resistance mechanism (HGT).

The genomic island carries various gene clusters responsible for pathogenicity and other functions of the enteric bacteria. The ubiquitous mechanisms of DNA exchange and a high rate of spontaneous mutation in bacteria are the major drivers of MDR. Biofilm is one of the common features of various bacterial strains that aid their survival under stringent conditions. These biofilms restrict the antibiotic from penetrating bacterial cells, and thus protect bacteria from the action of antibiotics, and facilitate the mechanism of resistance [50,55]. Sewage and wastewater treatment plants, hospital effluents, aquaculture, agriculture, and slaughterhouse wastes are the hotspots of genetic exchange [56]. Few external factors are also equally responsible for the enhanced antibiotic resistance, such as improper sanitation methods, unhygienic hospital settings, improper diagnostic, and therapeutic standards. Hospital-acquired community infection is one of the major concerns throughout the world. Hospital-acquired pathogens and co-infections have increased enormously. The hospital environment and the resistance gene reservoirs in the hospital facilitate the emergence and transmission of new modes of antibiotic resistance. Few bacteria harbor various resistance mechanisms (either intrinsic or acquired) that create those strains MDR [57]. Recombination events or HGT facilitate the transfer of genes from resistant strains to susceptible bacterial cells; genetic elements such as transposons, more precisely integron, are responsible for antibiotic resistance [12]. Some mechanisms are explained below, and Figure 2 schematically represents several mechanisms of antibiotic resistances such as increased drug efflux, decreased drug uptake, and drug or target modification/destruction.

#### 5.2.1. Antibiotic Modification or Degradation

Modification or degradation of antibiotics is one of the most common and important mechanisms of acquired resistance [22]. Bacteria contain genes or gene clusters that encode diverse antibiotic-modifying enzymes. These enzymes are effective against various classes of antibiotics such as aminoglycoside, chloramphenicol, and β-lactam [42]. Some of these enzymes are present in pathogenic strains, whereas other enzymes are present in both pathogenic and non-pathogenic strains. Enzymes can physically modify antibiotics, and they can actively decrease the concentration of drugs in the local environment. Therefore, it is a unique challenge to researchers and clinicians considering new approaches to anti-infective therapy [14,36].

#### 5.2.2. Antibiotic Efflux

Resistance mechanisms of various pathogens mainly involve permeability barriers for the active efflux of drug molecules [36]. Analysis of these mechanisms may be suitable targets for designing novel drugs. The synergistic action of reduced drug uptake and efflux is involved within the multiplicative action of the outer membrane permeability barrier, and it leads to the acquisition of high-level intrinsic and/or acquired resistance in many clinically important bacteria [42]. Ongoing trials targeted toward understanding the physical structures, function, and regulation of efflux systems will facilitate the exploitation of efflux pumps as new drug targets [22,50].

#### 5.2.3. Use of an Alternative Metabolic Pathway

Bacteria may also acquire antibiotic resistance by using alternate metabolic pathways [22]. A similar resistance mechanism was reported in small colony variants (SCVs) of *S. aureus*, the mechanism involved the activation of an alternative transcriptional program, leading to increased ATP production while retaining antibiotic resistance in SCVs. The growth rate of drug-resistant SCVs was also found to be high [58]. Similarly, bacteria can acquire the property of utilizing folate present in the environment rather than synthesizing folate [59].

#### 5.2.4. Reduction of the Inner and Outer Membrane Permeability

Porin mutations in resistant strains result in changes in membrane permeability and decrease drug uptake into the cell [42]. For example, in *P. aeruginosa* strains, OprD (specific porin) can cause mutation for carbapenem resistance. Moreover, reduction in permeability of the outer membrane may play an important role in conferring resistance to quinolones and aminoglycosides [60,61]. Alterations of porins could be achieved by three general processes: (1) a shift in the type of porins expressed, (2) a change in the level of porin expression, and (3) impairment of the porin function. Importantly, changes in permeability through any of those mechanisms frequently end in low-level resistance and are often related to other mechanisms of resistance, such as increased expression of efflux pumps [62].

## 6. Antibiotic Sequestration

The function of drug-binding proteins that inhibits the attachment of antibiotics with their target is defined as antibiotic sequestration. An example is the primary mechanism of resistance involved in producers of the bleomycin antibiotic family, where metal-bound or metal-free antibiotics are sequestered [30] by binding proteins, namely TlmA (in *Streptoalloteichus* *hindustanus* ATCC 31158) [63], BlmA (in *Streptomyces verticillus*) [64] and ZbmA (in *Streptomyces* *flavoviridis*) [63]. Protein-bound antibiotics in nearly every member of the bleomycin family are sequestered by one or more ABC transporter-related genes contained in the bleomycin biosynthesis gene cluster [30]. Similarly, a gene, *mrd*, cloned from *Streptomyces lavendulae* was reported to confer resistance to mitomycin C (MC) in both *Streptomyces lividans* and *E. coli*; characterization of the protein MRD further revealed that it specifically binds to the drug MC and prevents its activation. Furthermore, three immunity proteins, namely MlbQ, MlbY, and MlbZ, encoded in the lantibiotic NAI-107 biosynthetic gene cluster were found to contribute to immunity in the lantibiotic producer strain Microbispora ATCC PTA-5024 toward its antibiotic; the protein MlbQ was proposed to bind the cognate lantibiotic [33].

## 7. ESKAPE Pathogens

*Enterococcus faecium, Staphylococcus aureus, Klebsiella pneumoniae, Acinetobacter baumannii, Pseudomonas aeruginosa*, and *Enterobacter* spp. are the antibiotic resistant pathogens which are collectively known as ESKAPE. ESKAPE is responsible for causing huge infectious diseases [40]. Their ability to form biofilm is the best protection strategy against antimicrobial agents. The study of ESKAPE pathogens in clinical and non-clinical cases, their transfer of genetic material, resistance mechanism, and capability for biofilm formation can be the key for the development of new diagnostics and strategies. ESKAPE pathogens are important to study, as they show a high degree of drug resistance and can easily escape different therapeutic tools. They are responsible for nosocomial infection with high mortality rates. COVID-19 patients in the intensive care unit (ICU) often face secondary infection or co-infection due to ESKAPE pathogens [65]. These pathogens are responsible for the development of ventilator-associated pneumonia (VAP) [40]. Hospital equipment and surfaces require proper sterilization to overcome the infection caused by ESKAPE. Various research implies that antimicrobial peptides (AMPs), human milk oligosaccharides (HMOs), metal nanoparticles, antibacterial polymers, etc., show promising result against ESKAPE pathogens, but only in the laboratory system [66,67]. Modern approaches and specific drugs must be taken into account to fight ESKAPE pathogens. ESKAPE pathogens share a similar mode of drug resistant mechanisms like other bacteria, and they include drug inactivation, modification of target site, and reduction of antibiotic accumulation [67]. Apart from this, their biofilm forming ability on surfaces makes them more prone to drug resistance [66]. Genetic elements such as efflux pumps, integrons, transposons, and others are present in ESKAPE pathogens, which are responsible for their drug resistance capability. All of the ESKAPE pathogens are responsible for coinfection with SARS-CoV-2. However, A. baumannii showed a high level of drug resistance in the COVID-19 pandemic [40,65].

## 8. Global and Developing Countries Scenario

Antibiotic resistance is considered a global economic burden. In the year 2018, the Global Antimicrobial Surveillance System (GLASS), a part of the WHO, declared a huge list of resistant bacteria (*E. coli*, *K. pneumoniae*, *S. aureus*, and *Streptococcus pneumoniae* many more) widely spread among various countries. In this present scenario, several countries are presenting data regarding antibiotic surveillance to the WHO, while several face major problems in this surveillance program. The WHO is playing a significant role in promoting this study for the countries such as Kenya, Tunisia, Cambodia, Afghanistan, etc. GLASS can be considered a breakthrough to combat the global threat of antimicrobial resistance. Strong surveillance programs across different global and national locations may help in the enrichment of data and to reduce the burden of AMR [56,68]. Countries such as Pakistan, Thailand, Bangladesh, Nigeria, Nepal, and Brazil are facing huge antibacterial resistance due to various transmission sources including poultry, the environment, humans, etc. [68,69]. An anticipated death of around 3 million people by 2050 may be caused in continents like Asia, Africa, and North America due to antibiotic resistance. Creating awareness and educating people is the most prime focus of researchers/scientists for controlling the burden of antibiotic resistance. The WHO states that irrespective of the COVID-19 pandemic situation, many parts of the world are reporting a detailed surveillance site of AMR. All of this information covered by GLASS will provide a better opportunity to tackle health threats [68]. Apart from GLASS, the Global Antibiotic Research and Development Partnership (GARDP) and Interagency Coordination Group on Antimicrobial Resistance (IACG) are also working with the WHO for the improvement and development of antibiotics, and the implementation of global action plan to combat the present scenario. The WHO and governments of different countries can create awareness regarding hygiene maintain protocol among the common peoples. This safety measure along with knowledge of antimicrobial stewardship programs is very important in low- and middle-income countries [70].

Various measures should be adopted to tackle the problem of antibiotic resistance; however, the problem is persistent, and failure to combat the issue to date has been a matter of social, medical, and economic concern worldwide [42]. Even successful modern therapeutics would be impossible without the involvement of antibiotics. Thus, initiating significant research activities along with creating awareness and surveillance at the global level is urgently needed to resolve the issue. Very few new drugs are available in the market irrespective of the demand for new antibiotics [71]. The introduction of new antibiotics in the market of developing countries is very important to save the lives of a huge number of people [9].

## 9. Impact of COVID-19 Pandemic in AMR

With the exposure of the COVID-19 pandemic, the complication of drug resistance has increased, as the significant use of antibiotics in COVID-19 patients is a major area of concern [72]. For the treatment of COVID-19, the accelerated use of antibiotics, antiparasitic, and antiviral drugs has been performed [73], and this resulted in the spread of AMR. Initially, around 87.7% of COVID-19 patients were treated with antibiotics, whereas only 6.9% of patients were co-infected with bacteria or had a secondary infection. The unnecessary use of antibiotics must be reduced; increasing the rate of vaccination and other treatment options is necessary to combat COVID-19. In fact, few or a small number of COVID-19 patients require antibiotics for bacterial co-infection treatment, whereas a huge number of patients are receiving antimicrobials. Awareness and warnings regarding the increased rate of antimicrobial resistance with elevation in the case of COVID-19 patients all over the world must be put into immediate action. With a sudden outbreak of the COVID-19 pandemic globally, and due to the unavailability of effective medicines or vaccines, handling this situation is highly challenging, especially in the low- and middle-income countries [39,74]. Thus, the consumption of antibiotics and other antimicrobial agents has been increasing with the dissipation of COVID-19. In some cases, secondary bacterial infections were associated with this viral disease, and thus antibiotics were used to treat patients [75]. Initial studies by researchers [76,77], stated that COVID-19 patients were affected by different bacterial infections and thus antibiotic consumption was high. Thus, consumption of antibiotics by the COVID-19 patients without prior identification of bacterial infections must not be recommended and should be stopped immediately. Effective treatment procedures for COVID-19 patients are under research and successful analysis of the treatment options may enhance the use of appropriate and specific drugs [78,79]. According to WHO guidelines, the use of antibiotics for treatment of suspected or mild COVID-19 patients is not recommended without confirmed bacterial infection [39]. However, the use of antimicrobials for severe COVID-19 patients is used to combat the pathogens [78]. COVID-19 patients are hospitalized regularly worldwide, and they suffer from secondary or co-infections [75]. Thus, antibiotic therapy remains the best choice of treatment [80]. Hospital and ventilator-acquired pneumonia (HAP/VAP) was found in COVID-19 patients [10]. Usage of antibiotics in COVID-19 patients in the hospital at the early stage of this pandemic has created a burden in respect to antibiotic resistance [80]. Proper disposal of COVID-19 waste, appropriate antimicrobial policy, and multi-sectional approaches can help to reduce the impact of the COVID-19 pandemic and AMR in the environment.

## 10. Epidemiology and Surveillance

To some extent, the modern healthcare system is responsible for the transmission of community-acquired antibiotic resistance. Antibiotic resistance has become a worldwide alarming issue, due to low price, easy availability, and irrational use of antibiotic drugs [9]. Scientists and medical authorities, as well as government is focusing on alternative treatment options such as passive immunization, administration of antibodies, and phage therapy [14]. Government and Policymakers must ensure National Action Plan, maintaining databank and detail surveillance for stewardship of antibiotics and their resistance [81]. Dissemination of information, development of new drugs and vaccines are vital in the present situation. Vaccination of animals is recommended, and appropriate antibiotics administration must be considered for animal welfare [1]. Elaborative research and surveillance regarding antimicrobial resistance patterns, infectious pathogen transmission, and their appropriate treatment options are of great importance to fight against COVID-19 pandemic and antimicrobial resistance [10,72].

### 10.1. Control Strategy of Antibiotic Resistance

Appropriate indication, dosage, means, and time of drug intake strictly according to medical advice is a necessary component for controlling antibiotic resistance. In addition, antibiotic resistance profiling of patients is recommended before prescribing antibiotics [82]. Moreover, strict implementation of infection control measures, proper hygiene and sanitation standards, and appropriate disposal or discharge of medical wastes must be ensured in hospitals and communities. All these control strategies must be regulated by strict government policies to ensure their implementation by the common people. Researchers believe that the application of computational biology in combination with nanotechnology can open new avenues to combat the problem of antibiotic resistance [42]. Pharmaceutical industries are currently aiming to develop new drugs that can fight against new resistant strains [14]. However, with the development of improved and novel pharmaceutical products, bacteria are also expanding their resistance to different avenues by modifying their molecular regulators. Knowingly or unknowingly, people in every corner of the world are excessively using antibiotics without proper medical concern. Furthermore, to overcome the issues of antibiotic resistance, the government must release ample funds as well as set new policies. Personalized medicine and appropriate antibiotic therapy for patients can be novel interventions for antibiotic resistances.

At an individual level, we can put some of our effort to combat the expansion of antibiotic resistance by using antibiotics only prescribed by doctors, restricting of overuse of antibiotics in the food and animal sector, and maintaining hygiene protocol. Control in antimicrobial resistance is important to mandate to overcome or reduce the impact of COVID-19. Management practices and strategies must be taken into account for restricted use of antimicrobials and biocide to tackle the problem of AMR, public health, and the environment. Encouraging hygiene measures but discouraging overuse of disinfectants and antimicrobials must be a scheme to overcome the problem of AMR along with the COVID-19 pandemic. Prescribing antibiotics, their proper use and behavioral change of people towards health management issues can be a strategy to fight antibiotic resistance [6,39]. Creating AMR awareness among the population of the developing countries must be undertaken together by the policymaker, researchers, and implementation teams [83]. With the advent of COVID-19, few hygienic measures such as hand washing, proper disinfection, and social distancing have developed among people of the world [39]. All of these measures in the near future will definitely help to reduce the spread of AMR. Along with this, the surveillance on use of antimicrobial agents, susceptibility profiling of bacterial infection, and the development of therapeutic measures for COVID-19 will help to focus on controlling the spread of both viral and bacterial infection [82].

### 10.2. Therapeutic Interventions

Combating Antibiotic-Resistant Bacteria Biopharmaceutical Accelerator (CARB-X) (https://carb-x.org/), the Replenishing and Enabling the Pipeline for Anti-Infective Resistance (REPAIR) Impact Fund (https://www.repair-impact-fund.com/), Global Antibiotic Research and Development Partnership (GARDP) have taken initiatives to develop new antimicrobials and other treatment options to encounter the problem of AMR [10,40,84]. According to the WHO pipeline report (2020), the new antibiotics are not sufficient to handle the rise of antimicrobial resistance. Application of different therapies including antibody or bacteriophage rather than antibiotics is suggested and recommended by WHO. For the development of an innovative and promising product to strengthen and accelerate the drive of antibiotic research AMR Action Fund and Antimicrobial Resistance Multi-Partner Trust Fund (AMR MPTF) was established. Surveillance systems in different countries of the world by GLASS will help to develop a new therapeutic intervention. In this pandemic situation, new normal living such as social distancing, reduced travel, and increased personal hygiene is the key to better lifestyle [81]. The development of mRNA vaccines and new diagnostic tools would be very effective [39]. New therapeutic intervention is important for carbapenem-resistant *Enterobacteriaceae* and *A. baumannii*. Secondary plants metabolites, probiotics, prebiotics, and others may be better alternative approaches for the treatment of diseases [15,85,86].

Focusing on identifying the gene/s responsible for resistance, followed by their detailed molecular analyses will help combat the problem. Thus, the accessibility of available information on bacterial genomes along with functional data for various antibiotic resistance genes will help in developing efficient preventive tools and diagnostic pathways. Culture-based identification techniques for bacterial isolates followed by antimicrobial susceptibility profiling are being extensively employed. However, these culture-based assays are associated with several drawbacks, such as their time-consuming nature and inaccurate interpretation. Since accurate and on-time analysis of the AMR profile is crucial, molecular approaches are considered suitable because of their superior sensitivity and specificity, rapidity, and ability to identify bacterial drug susceptibilities. Molecular tools can identify specific resistance mechanisms and aid in selecting the most effective antibiotics for patients. Molecular-based diagnostic tools can also reduce healthcare costs. Moreover, the speed and sensitivity of molecular-based approaches provide benefits in terms of time and accuracy. The extensive molecular characterization of antibiotic resistance genes is urgently required to develop novel therapeutic strategies for preventing life-threatening infections [87].

Gram-negative bacterial pathogens are at the most alarming state as they are becoming resistant to nearly every drug to some extent. Therefore, studying the transmission or spread of MDR gene/s is vital for analyzing transmission pathways and risk factors for infection. One of the most important challenges is to precisely identify the drug-resistant for a patient in a short period time and cost-effective manner. Since the tracking of various resistance mechanisms or gene/s responsible for resistance is important, proper database and diagnostic techniques should be maintained, and wise use of antibiotics by improving its prescribing/stewardship should be emphasized [10,82].

## 11. In Silico Analysis

In silico analysis for research regarding antimicrobial resistance will open a new avenue for scientists and the pharmaceutical industry as well as policymakers [88]. Molecular docking and homology modeling of the available drugs can be easily investigated [69,89]. In silico study will also help in understanding the drug resistant mechanism, prediction of traits responsible for AMR, protein-protein interaction, changes in structural conformation and analysis of mutational hotspot region [90]. Apart from this bioinformatics tools and software will help in the analysis of serogroup, antigenic profiling, identification of plasmid and antibiotic resistance genes [22]. Application of metagenomics, antibiotic resistance databases, and machine learning tools will help to predict the resistance pattern and profile [22]. Therefore, all this information can be useful in research related to AMR and its treatment options. Whole-genome sequencing (WGS) and/or next-generation sequencing (NGS) should be effective for surveillance of AMR gene in bacteria and this information will guide in the intervention of AMR [68,90,91]. Different web-based tools or software can be developed for easy and rapid detection of determinants or genetic elements responsible for MDR [2]. Transcriptomic and metagenomics studies are emerging fields of advanced science, therefore using these techniques for drug discovery and curbing antimicrobial resistance is the forte. Personalized drugs can also be designed using in silico approaches [22].

## 12. Conclusions

Irrespective of the measures taken by the WHO and the CDC, many people in different parts of the world are not aware of antibiotic resistance and its consequences. The problem is not only limited to the healthcare sector, but also an increasing thrust for the world economy. With the emergence of MDR, PDR and other types of resistant bacteria globally, treatment option in medical science is facing various hurdles. The shortage of new antimicrobial agents is a major concern in healthcare systems. Therefore, immediate enforcement of epidemiological and surveillance studies is required for the analysis and prevention of antibiotic resistance. Sufficient knowledge and information can be the key to controlling MDR. Strict government policies and regulations throughout the world are also essential for monitoring the use of antibiotics and other related aspects. Finally, multidisciplinary approaches must be considered to eliminate the serious threat caused by MDR. Collaborative studies of computational biology with nanotechnology can open new avenues to combat the problem of antibiotic resistance. New knowledge on MGEs could enhance understanding of the genesis of variants and pathogen evolution. It may also help in developing potential therapeutic interventions for pathogen spreading and for restricting the growth of pathogenic strains. Additionally, a model for decreasing antibiotic consumption can be developed by knowing the transmission rate of antibiotic resistance from animals to humans.

The goal of the authors is to provide everyone with some basic ideas regarding the appropriate usage of any antimicrobial agents. This study has focused on the different aspects of antibiotic resistance and its associative problem with public health. Few conceptions of molecular mechanisms of antibiotic resistance and emergence of antibiotic resistant superbugs were discussed to provide some insights for the formulation of new treatment options. The authors also tried to outline the global impact of the COVID-19 pandemic on antibiotic resistance. The review provides an overview regarding the importance of antibiotic resistance from an enlarged perspective, its arising challenges and multi-sectional strategy that may help the government, researchers, and other people to tackle this vital issue.

## Figures and Tables

**Table 1:** Different mode of action of antibiotics and its resistance mechanisms along-with few bacterial examples.

Antibiotics	Mode of Action	Mechanism of Resistance	Examples	Reference
ß-Lactams (Cephalosporin, Carbapenems, etc.)	Peptidoglycan biosynthesis Cell wall synthesis Inhibition	Hydrolysis, efflux, altered target, reduced permeability, inactivation of antibiotics via ß-lactamase (extended spectrum ß-lactamase; carbapenem-hydrolyzing ß-lactamase)	*Staphylococcus aureus, Pseudomonas aeruginosa*, Enteric bacteria, *Streptococcus pneumoniae*, *Vibrio cholerae, Escherichia coli, Klebsiella pneumoniae, Acinetobacter baumannii*	[12,30,42,43,44,45]
Aminoglycosides (Gentamicin, Streptomycin, Spectinomycin, Amikacin, Tobramycin, etc.)	Inhibition of Translation and cell membrane synthesis	Modifying enzyme inactivation by Phosphorylation (phosphorylase), acetylation (acetylase), nucleotidylation, efflux, altered target ribosomal binding site, decrease uptake by reducing permeability, other modifying enzymes includes acetyltransferases, adenyl transferases, phosphotransferases.	Enteric bacteria, Staphylococci, Streptococci, Bacteriodes, *Pseudomonas aeruginosa*, *Vibrio cholerae, Escherichia coli, Klebsiella pneumoniae, Acinetobacter baumannii, etc.*	[12,30,42,43,44,45]
Glycopeptides (Vancomycin, Teicoplanin)	Peptidoglycan biosynthesis	Altered target	Enterococci, Lactobacilli, *Staphylococcus haemolyticus*, *Enterococcus* *faecium,* *Enterococcus* *faecalis, etc.*	[30,42,43,44,45]
Tetracyclines (Tigecycline, Minocycline, Doxycycline)	30S ribosomal subunit	Monooxygenation, ABC efflux pump, ribosomal modification, tetracycline inactivating enzyme	Staphylococci, Streptococci, Enterococci, Enterobacteriaceae, Haemophilus, Listeria, *Acinetobacter baumannii,* etc.	[30,42,43,44,45]
Macrolides (Erythromycin, azithromycin)	Translation	Glycosylation, efflux, methylation of rRNA target	Streptococci, Enterococci, Staphylococci, *Acinetobacter baumannii*, etc.	[12,43,45]
Phenicols (Chloramphenicol, Azidamphenicol, Thiamphenicol)	Translation inhibitors	Acetylation by chloramphenicol acetyltransferase, efflux pump, target site alteration	*Bacillus subtilis, Streptococcus pneumoniae*, Enterobacteriaceae, *Haemophilus influenzae*, *Vibrio cholerae, Escherichia coli* etc.	[12,42,43,44,45]
Folate inhibitors (Trimethoprim, Sulfamethoxazole)	Inhibit folate synthesis pathways	Efflux, altered target	Staphylococci, Streptococci, Enterobacteriaceae, Neisseria, *Acinetobacter baumannii*, etc.	[30,45]
Rifamycins (Rifampin)	Transcription	ADP-ribosylation, efflux, altered DNA-dependent RNA target	Enteric bacteria, Staphylococci, Streptococci, *Mycobacterium tuberculosis*, *Vibrio cholerae, Escherichia coli, Klebsiella pneumoniae, Acinetobacter baumannii*, etc.	[12,30,45]
Quinolone (nalidixic acid, ciprofloxacin, levofloxacin, ofloxacin, norfloxacin)	Inhibitors of DNA synthesis Topoisomerase I and II	Altered DNA gyrase or DNA topoisomerase IV subunit A (parC) efflux or reduced permeability	*Staphylococcus aureus, Pseudomonas aeruginosa, Staphylococcus epidermis*, *Streptococcus pneumoniae* *Acinetobacter baumannii* etc.	[12,30,42,43,44,45]
Cationic peptides (Colistin, Polymyxin-B)	Cell membrane Lipopolysaccahride layer of bacteria	Altered target, efflux	*Escherichia coli, Salmonella Typhimurium*, *Acinetobacter baumannii*, etc.	[30,42,45]
